# Microbial profile along the maternal-infant axis: Early characterization and relationships in breastfeeding women and their newborns in a Colombian population

**DOI:** 10.1371/journal.pone.0340091

**Published:** 2026-01-08

**Authors:** Diana Carolina Londoño-Sierra, Claudia Jaramillo-Mazo, Shadia Blel, Victoria Mesa, Sandra Lucia Restrepo-Mesa, Julián Paul Martínez Galán, Laura Sierra-Zapata

**Affiliations:** 1 Research Group on Human Food and Nutrition, School of Nutrition and Dietetics, University of Antioquia UdeA, Street 70 No. 52-21, Medellín, Colombia; 2 Biosciences and Technology (TechLife) Research Group, School of Applied Sciences and Engineering, University EAFIT, Medellín, Colombia; 3 Physiopathologie et Pharmacotoxicologie Placentaire Humaine Microbiote Pré & Postnatal (3PHM), INSERM, UMR-S 1139, Université Paris Cité, Paris, France; Washington State University - Spokane, UNITED STATES OF AMERICA

## Abstract

**Objective:**

To characterize the maternal-infant microbiota and examine microbial relationships among the maternal gut, human milk, and infant gut microbiomes in a cohort of Colombian mother-infant pairs at a single time point within the first three months postpartum.

**Study design:**

A cross-sectional study was conducted with 30 mother-infant pairs. A total of 90 samples of human milk, maternal feces, and infant feces from healthy, exclusively breastfeeding pairs were analyzed to assess bacterial composition and diversity using microbiota analysis performed with 16S ribosomal RNA (rRNA) sequencing on the Illumina platform.

**Results:**

The maternal gut microbiota was dominated by *Subdoligranulum* spp., *Akkermansia* spp., *Christensenellaceae R-7 group*, and *Bacteroides* spp., while the human milk microbiota was dominated by *Streptococcus* spp. and *Staphylococcus* spp. In contrast, the infant gut microbiota was primarily made up of *Escherichia-Shigella* spp. and *Bifidobacterium* spp. A total of 644 ASVs were shared among the maternal gut microbiota, human milk, and infant gut microbiota.

**Conclusions:**

This study offers the first comprehensive characterization of the maternal-infant microbial axis in a specific Colombian population, highlighting distinct microbial profiles in healthy lactating mothers, human milk, and their infants, and indicating potential microbial interactions that could be important for early-life colonization.These findings should be considered in clinical and nutritional care strategies for lactating women, as they may provide an opportunity to promote health in the mother-infant dyad. Future studies will need to investigate factors, including nutritional factors, which may influence the microbiota along the maternal-infant axis.

## Introduction

The development of the human microbiota in early childhood is crucial for long-term health outcomes [1]. During this period, the microbiome is highly adaptable and plays an important role in establishing digestive and immune infant health [2]. It acts as a central point for interactions with other physiological systems throughout life and helps prevent significant health conditions in children associated with dysbiosis signatures [3,4]. It has been documented that approximately one-quarter of the infant gut microbiota may originate from the microbial transfer via human milk [5]. Species-level studies have identified that certain bacteria coated with secretory immunoglobulin A (sIgA), such as *Escherichia-Shigella* spp., as well as uncoated bacteria like *Pseudomonas* spp., and species of *Bifidobacterium* spp. (*B. longum, B. adolescentis, B. bifidum, and B. breve*), may be transferred from the maternal gut to human milk and subsequently to the infant [6,7]; an overlap of genera such as *Bifidobacterium* spp., *Escherichia-Shigella* spp., *Bacteroides* spp., *Streptococcus* spp., and *Veillonella* spp. has been identified [8,9].

The origin of milk-associated bacteria has been linked to colonization from various sources, including an endogenous route called the entero-mammary pathway [10]. This connection highlights the importance of characterizing the gut microbiota of lactating women, especially since pregnancy involves microbiome changes related to metabolic adaptations during this period [11]. However, despite the increasing knowledge about the gestational microbiome, the gut microbial composition of lactating women remains an underexplored area of research. The available evidence shows that during the postpartum period, the intestinal microbiota of women is characterized by the phylum Firmicutes with the dominant families being *Lachnospiraceae*, *Clostridiaceae*, *Streptococcaceae* and *Ruminocococcaceae*, and the dominant genus *Bacteroides* spp. [12].

Despite its importance, knowledge about microbiota dynamics during the first 1,000 days of life remains limited in the Latin American context. Studies in Brazil [13] and Colombia [14]. have focused on characterizing human milk microbiota, and research in Mexico has looked at maternal-to-infant bacterial transfer [15]. However, to our knowledge, no studies in Colombia have simultaneously analyzed maternal intestinal microbiota, human milk, and infant intestinal microbiota during the first three months of life, a period of particular importance due to the biological succession involved in microbiome maturation, which may be linked to the early development of diseases such as allergies, obesity, and diabetes [16–18].

Understanding the microbiota and its relationship along the maternal-infant axis in healthy populations is essential for elucidating the biological mechanisms that could support targeted interventions in food, health, and nutrition, particularly among lactating women, who often lack comprehensive nutritional follow-up. In this context, the present study aimed to characterize the maternal gut, human milk, and infant gut microbiota, and to explore the microbial relationships among these ecosystems in a cohort of Colombian mother-infant dyads at a single time point within the infant’s first three months of life.

## Methods

### Study design and participants

A cross-sectional study was conducted with 30 mother-infant pairs from Colombia during the first trimester of breastfeeding, selected conveniently. The recruitment period was from June 16, 2023, to June 30, 2024. Inclusion criteria for lactating women were age between 19 and 39 years, singleton pregnancy, absence of diseases or complications during pregnancy and postpartum (such as anemia or diabetes), body mass index (BMI) within the normal or overweight range, low physical activity, household food security, full-term delivery, and exclusive breastfeeding at the time of evaluation. Women were excluded if they had obesity (BMI > 30 kg/m²), underweight (BMI < 18.5 kg/m²), or if they had taken medications, antibiotics, laxatives, proton pump inhibitors, or probiotic supplements chronically or within 30 days before sample collection. Basic medical history, anthropometric characteristics, and fecal samples were collected.

For the recruitment of participating women, the medical records were first reviewed to obtain information on maternal health status. Subsequently, potential participants were contacted through the health institutions to verify the eligibility criteria and to schedule a visit for signing the informed consent. During this visit, biological samples were collected, anthropometric measurements (weight and height) were taken to calculate BMI, and the Latin American and Caribbean Food Security Scale (ELCSA) was administered to verify household food security, understood as the condition in which all people, at all times, have physical, social, and economic access to sufficient, safe, and nutritious food that meets their daily energy requirements and food preferences for an active and healthy life [19].

### Sample collection and storage

The samples were collected at a single time point between 15 days and 3 months postpartum. Infant fecal samples were collected directly from diapers using sterile containers. For maternal fecal collection, each participant was provided with a sampling kit that included a sterile container, gloves, two sealed plastic bags, and alcohol, along with detailed instructions. A trained nutrition and dietetics professional manually collected human milk samples in the early morning hours, from the breast opposite to the last nursing session or the breast not being used for breastfeeding at the time of collection. The nipple and areola were cleansed with sterile gauze and 0.5% chlorhexidine. The first drops of milk were discarded, and between 15–20 mL were collected into sterile, RNase- and DNase-free tubes (Corning Incorporated, Corning, NY, USA). All samples were transported in a portable cooler with dry ice and stored at –80°C until analysis.

### DNA extraction, quantification, and sequencing

Total genomic DNA was extracted using the GeneJET Genomic DNA Purification Kit (Thermo Scientific, USA), following the manufacturer’s instructions with specific adaptations according to sample type. For fecal samples, a pre-wash with phosphate-buffered saline (PBS) was performed before extraction, using the resulting pellet. DNA quantification was carried out by measuring the OD260/280 ratio using UV absorbance with a NanoDrop spectrophotometer (Thermo Scientific, Wilmington, USA). Amplification of the V3–V4 hypervariable regions of the 16S ribosomal RNA (16S rRNA) gene was performed using 1 µL of DNA (approximately 25 ng on average). Total genomic DNA was extracted using the GeneJET Genomic DNA Purification Kit (Thermo Scientific, USA), following the manufacturer’s instructions with specific adaptations according to sample type. For fecal samples, a pre-wash with phosphate-buffered saline (PBS) was performed before extraction, using the resulting pellet. DNA quantification was carried out by measuring the OD260/280 ratio using UV absorbance with a NanoDrop spectrophotometer (Thermo Scientific, Wilmington, USA). Amplification of the V3–V4 hypervariable regions of the 16S ribosomal RNA (16S rRNA) gene was performed using 1 µL of DNA (approximately 25 ng on average). The primers used were Bakt_341F: CCTACGGGGGNGGCWGCAG and Bakt_805R: GACTACHVGGGGGTATCTAATCC, and each sample was assigned a unique 6-base pair (bp) barcode. Barcoded PCR products were purified from triplicate reactions with an agarose gel band purification kit (Illustra GFX PCR dna and gel Band Purification Kit, GE Healthcare,UK). Equimolar concentrations of PCR amplicons were quantified by fluorometric methods (Qubit 3.0—Thermo Fisher Scientific,Waltham, MA, USA). Purified amplicons were pooled in equimolar amounts (~50 ng per sample) for library preparation). Sequencing was conducted on the Illumina MiSeq platform using paired-end technology (2 × 300 bp), generating approximately 100,000 reads per library. Sequencing services were provided by Macrogen (Seoul, South Korea).

### Bioinformatic analysis

A total of 90 samples, including maternal feces, infant feces, and human milk, were analyzed to characterize their bacterial communities. Raw demultiplexed paired-end reads were inspected with FastQC and processed in QIIME2 version using the DADA2 plugin for denoising [20,21]. This method involved quality filtering, dereplication, merging of paired end reads, and chimera removal. Forward and reverse reads were truncated at 260 bp and 230 bp, respectively, with no trimming at the 5’ ends. The QIIME2 feature-classifier plugin was used with the Vsearch alignment method to classify these sequences according to their taxonomic information [22]. Amplicon sequence variants (ASVs) were inferred and compiled into a feature table. Taxonomic assignment was performed using the consensus BLAST method against the SILVA v138 reference database at a 99% sequence identity [23]. A rarefaction depth of 3,950 reads per sample was applied to normalize sequencing effort across samples and prevent diversity biases. The data generated were exported for analysis in Rstudio v4.1.0 [24].

### Data analysis and statistics

Absolute and relative abundances of the identified taxa were estimated for each sample group based on the generated ASV table. Alpha diversity and richness were assessed using the Chao1 and Shannon indices [25,26]. Group comparisons were performed using the non-parametric Kruskal–Wallis test. A non-metric multidimensional scaling (NMDS) analysis was carried out on the pairwise Bray–Curtis dissimilarities between bacterial community composition within and between mother, milk, and infant, ensuring that stress values indicate a good fit. A Permutational multivariate analysis of variance (PERMANOVA) was applied using the Adonis2 function (vegan: adonis2). Differential abundance of bacterial taxa across ecosystems was assessed using the ANCOM-BC method (Analysis of compositions of microbiomes with bias correction), implemented via the ANCOMBC package in R software (version 4.1.0) [24]. The model included sample type (maternal feces, human milk, infant feces) as a covariate, and we included genera that had a relative abundance ≥1% in any of the ecosystems, in addition we evaluated whether the type of delivery had a response in the bacterial community, without identifying differences, so it was not included as a covariate (S1 Fig). Results of differentially abundant taxa were visualized using a heatmap. Statistical significance was defined as a p-value < 0.05.

This study was conducted by the ethical principles outlined in the Declaration of Helsinki. All participating women provided written informed consent. The study protocol was approved by the Bioethics Committee of the Faculty of Dentistry at the University of Antioquia, Colombia (Approval No. 151–2023, Minute No. 05 of 2023).

## Results

### Genomic data counts and quality

The number of inputs reads per sample ranged from 64,547–148,449, with a mean of 101,600 (± 16,121). After quality filtering, an average of 80.7% of reads were retained (mean = 82,014 reads), and 80,241 reads per sample were successfully denoised. Following chimera removal, an average of 74,501 non-chimeric reads were retained, representing 73.5% of the initial input. The percentage of chimeric sequences detected varied between 4.45% and 23.77% across samples, with a mean of 12.5%. These results indicate high sequencing quality and efficient noise removal throughout the dataset [20] (S1 Table).

### Characteristics of the mother-infant dyads

We characterized the mature breast milk and intestinal microbiota of 30 women and their healthy infants during the first trimester postpartum. The participating women had a mean age of 25 ± 6 years and were classified as Food and Nutrition Secure according to the Latin American and Caribbean Food Security Scale (ELCSA). At the time of evaluation, the average maternal body weight was 60.8 ± 0.9 kg and average height was 157 ± 0.048 cm, with 60% presenting an adequate Body Mass Index (BMI). Regarding the infants, 77% were delivered vaginally, with a mean age of 42 ± 21 days. The average birth weight was 3299 ± 275 g, and the mean birth length was 49.8 ± 1.7 cm (Table 1).

**Table 1:** Gestational and anthropometric characteristics of mother-infant dyads.

Variable	n	%
**Previous pregnancies**		
No pregnancies	13	43.3
1-2	16	53.3
≥3	1	3.3
**Iron Supplementation**		
Yes	30	100
No	0	0
**Acid Folic Supplementation**		
Yes	27	97
No	1	3
**Type of delivery**		
Vaginal	23	76.7
Cesarean section	7	23.3
**Newborn sex**		
Female	11	36.7
Male	19	63.3
**Breastfeeding in the first hour**		
Yes	26	86.7
No	4	13.3
**Anthropometric**		
**Birth Weight (g)**		
2500 – < 3000	3	10
3000–3500	20	66.7
>3500 −4000	7	23.3
**Length at birth (cm)**		
≥50	18	60
<50	12	40
**Breastfeeding BMI (Kg/m**^**2**^)		
Adequate	18	60.0
Overweight	12	40.0

Data presented as sample (n) and %. IBM: Body Mass Index.

### Infant gut, milk, and maternal gut microbiota differ significantly, each dominated by distinct taxa

**Fig 1:**
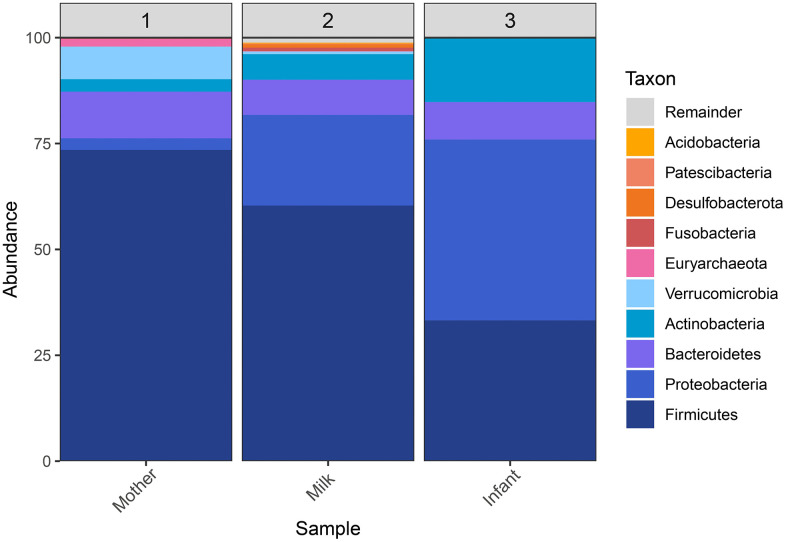
Bacterial composition microbiota in the maternal-infant axis.

The main bacterial phyla and genera were identified in each sample. At the phylum level, the relative abundance of Proteobacteria (46.2%) and Actinobacteria (16.7%) was higher in the infant gut microbiota compared to human milk (20.9% and 5.6%, respectively) and the maternal intestinal microbiota (2.4% and 3.3%, respectively). In contrast, Firmicutes were predominant in the maternal intestinal microbiota (73.6%) (Fig 1).

At the order level, Oscillospirales (36.4%) was representative of the maternal gut microbiota; *Lactobacillales* (27.5%) was characteristic of human milk, while *Enterobacterales* (41.7%) dominated the infant gut microbiota. At the genus level, approximately 50% of the infant microbiota was composed of *Escherichia-Shigella* spp. and *Bifidobacterium* spp. In human milk, dominant genera included *Streptococcus* spp., *Staphylococcus* spp., *Bacteroides* spp., and *Corynebacterium* spp. The maternal intestinal microbiota was dominated by *Subdoligranulum* spp., *Akkermansia* spp., *Christensenellaceae_R-7_group*, *Bacteroides* spp., *Oscillospiraceae UCG-002*, *Agathobacter* spp., *Ruminococcus* spp., *Faecalibacterium* spp., and *Blautia* spp.

### Infant gut microbiota is less diverse than human milk and maternal gut microbiota

**Fig 2:**
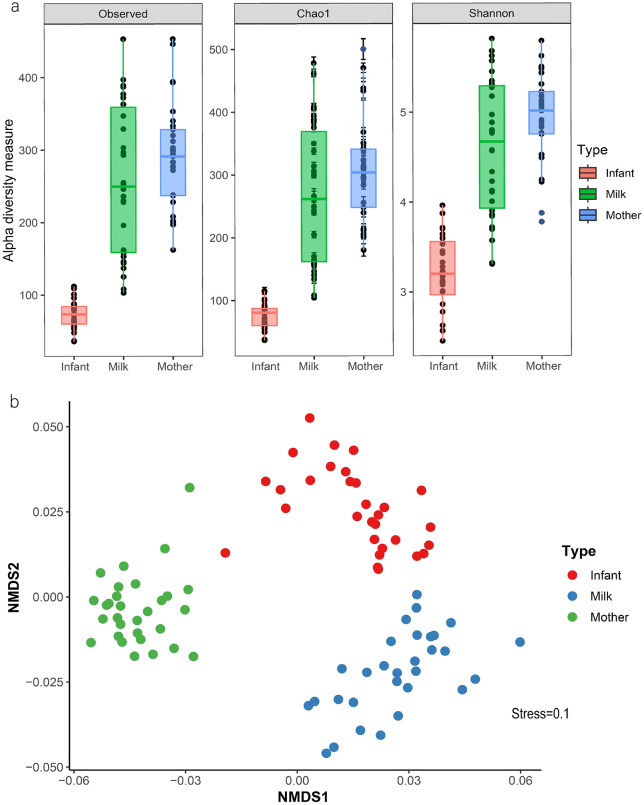
Diversity of microbiota composition across maternal-infant axis. **a)** Alpha diversity measured using the Shannon and Chao1 indices. **b)** Non-metric multidimensional scaling (NMDS) plot illustrating the median compositional beta diversity.

Compared to human milk and maternal gut microbiota, the infant gut microbiota exhibited significantly lower observed alpha diversity, estimated species richness, and overall richness (p < 0.001). However, no significant differences were found between the maternal and milk microbiota in these indicators (Shannon diversity and Chao1 richness index; p > 0.182) (Fig 2a). NMDS based on differences in relative microbial composition was performed to explore the similarity among ecosystems. This analysis revealed that beta diversity significantly differed across groups (PERMANOVA p = 0.001) (Fig 2b).

### *Bifidobacterium* spp. and *Escherichia-Shigella* spp. differ between milk and infant microbiomes

**Fig 3:**
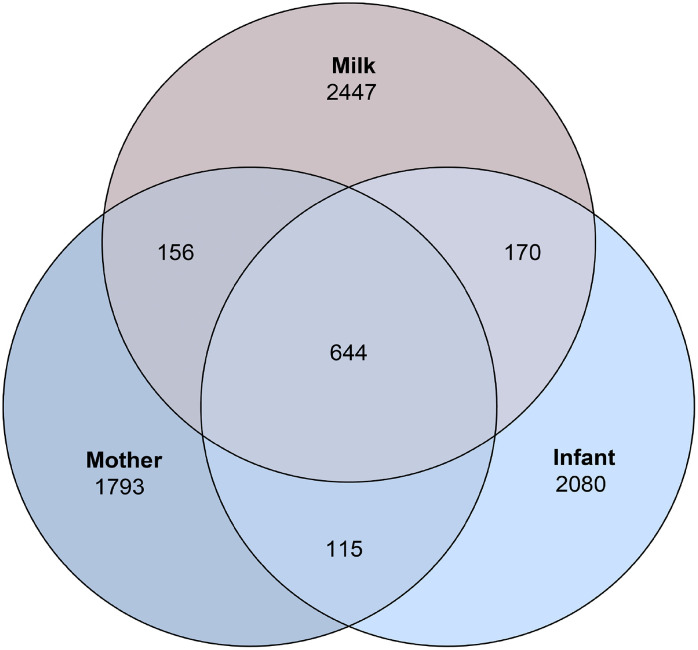
Venn diagram. Number of common and unique Amplicon Sequence Variants (ASVs) between the samples.

We explored the potential microbial transmission between sample types by assessing the number of ASVs. A total of 644 ASVs were found to be common across the maternal gut, human milk, and infant gut microbiota (Fig 3). Additionally, 800 ASVs were shared between the maternal microbiota and human milk, 814 between human milk and the infant, and 759 between the mother and the infant. Among these shared ASVs, we identified members of the orders *Bifidobacteriales, Enterobacterales, Lactobacillales, Erysipelotrichales, Staphylococcales, Clostridiales,* and *Lachnospirales*.

Subsequently, we evaluated the differential abundance of dominant bacterial phyla. Firmicutes and Bacteroidetes were significantly more abundant in the maternal gut microbiota compared to both human milk and infant microbiota (p = < 0.001). However, the relative abundance of Firmicutes did not differ significantly between milk and infant samples (p = 1). In contrast, Proteobacteria exhibited significant differences in abundance across the three sample types (p= ≤ 0.001), whereas Actinobacteria, Fusobacteria, and Patescibacteria showed no significant variation (p = 1) (S2 Fig).

**Fig 4:**
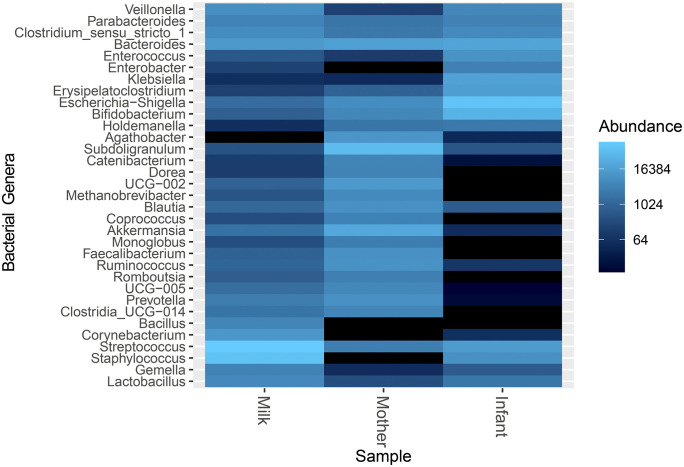
Differentially abundant bacterial genera across maternal-infant axis.

At the genus level, four genera *Bacteroides* spp., *Erysipelatoclostridium* spp., *Parabacteroides* spp., and *Akkermansia* spp. did not show significant differences (p > 0.05). *Bifidobacterium* spp. and *Escherichia-Shigella* spp. were not significantly different between maternal and milk samples (p = 0.07 and p = 0.88, respectively). Still, they were significantly different between milk and infant samples, as well as between maternal and infant samples (p < 0.001) (Fig 4).

## Discussion

The findings of this study reveal that, in this group of Colombian lactating women and their newborns, the different microbiotas along the maternal-infant axis during the first trimester show phylogenetically distinct patterns. Among this microbial diversity, specific taxa are shared across the samples. Among this microbial diversity, specific taxa were shared across samples. These findings are consistent with studies conducted in other settings, which propose an important biological pathway for the modulation of the microbiome during the first 1,000 days of life [8,27,28,29,30].

In the composition of the maternal gut microbiota, we identified Firmicutes as the dominant phylum, consistent with previous studies [12], including research conducted in Mexican women [30]. However, at the genus level, our findings differed from those reported in other investigations, such as those by Butts et al. (Manawatu–Wanganui region, New Zealand) [31]. Williams et al. (USA) [28] and Juárez-Castelán et al. (Mexico) [32]. In this cohort of healthy Colombian lactating women, *Subdoligranulum* spp., rather than *Bacteroides* spp., was the dominant genus in the maternal gut microbiome, followed by *Akkermansia* spp., both of which have been reported to occur at lower abundances in similar populations [31–34].

These genera are associated with favourable metabolic and physiological effects. *Subdoligranulum* spp. is linked to butyrate production and is phylogenetically related to *Faecalibacterium* spp., a taxon consistently associated with a favourable metabolic profile [35,36]. *Akkermansia* spp. contributes to epithelial barrier protection, supports maintenance of the mucus layer, and improves metabolic disorders (such as diabetes) through multiple mechanisms, including stimulation of glucagon-like peptide-1 (GLP-1) secretion by intestinal L cells, which promotes insulin production [37,38]. Additionally, both *Akkermansia* spp. and the *Christensenellaceae* R-7 group have been associated with improved cardiovascular health [39,40]. Based on these observations, we hypothesize that the microbial profile identified may support adaptation to the new biological environment characteristic of the postpartum period, which follows pregnancy-related physiological and metabolic changes such as reduced insulin sensitivity and gestational hyperlipidemia [11]. However, this hypothesis requires confirmation through additional longitudinal studies.

These changes may support postpartum recovery and facilitate adaptation to a new biological environment following the physiological, endocrine, metabolic, and microbial shifts associated with pregnancy [11]. Although dietary variables were not assessed in this study, previous evidence [41] suggests that our findings may also be influenced by maternal factors such as nutritional status and dietary habits. Nevertheless, all participating women were neither malnourished nor obese, had adequate food availability at home, and presented no health conditions known to alter the microbiota. These favourable conditions may have contributed to the establishment of a metabolically advantageous gut microbiota during the early postpartum period.

Maternal microbial ecosystem plays a key role in shaping both the human milk microbiome and the infant gut microbiota. Bogaert et al. [27] demonstrated that 58.5% of the infant microbiota originates from various maternal niches, with human milk identified as the main route of microbial transfer. In our study, we observed the superposition of ASVs between mother, human milk, and infant ecosystems, which aligns with the entero-mammary pathway [10]. We also observed ASVs shared only between the mother’s gut microbiota and milk but not transmitted to the infant, as well as some ASVs shared between human milk microbiota and the infant gut microbiota but not originating from the mother, findings consistent with those of Meng et al.[8], in a cohort of Chinese women, a superposition was observed between maternal gut microbiota and breast milk microbiota, as reported by Shama et al.[42], between human milk and gut microbiota of infants with low birth weight. This could suggest selective transmission of bacteria from mother to infant through milk and other sources of colonization for the infant.

Within the maternal-infant microbial axis, human milk plays an intermediary role in the transmission of prebiotic compounds and microorganisms that contribute to the infant’s gut colonization. It is estimated that an infant consuming 800 mL of breast milk per day receives approximately 10⁷ to 10^8^ of bacterial cells [43]. In this study, the dominant phyla identified in human milk were Firmicutes and Proteobacteria, consistent with findings reported by Oddi et al. [44] in breast milk from Argentine women. Additionally, representative genera included *Streptococcus* spp., *Staphylococcus* spp., *Bacteroides* spp., and *Corynebacterium* spp. Some of these genera are known as common residents of the skin microbiome [43].These findings highlight the importance of gaining a deeper understanding not only of the maternal gut microbiota but also of the microbial communities inhabiting other maternal body niches. Such knowledge may open future opportunities for targeted modulation or therapeutic strategies aimed at protecting the skin microbiome of the mammary gland, thereby preventing dysbiosis that could interfere with breastfeeding and disrupt the natural microbial transfer to the newborn.

In the evaluated infants, the microbial community was dominated by the phyla Proteobacteria, Firmicutes, and Actinobacteria. These findings are consistent with those reported by Corona-Cervantes et al. [15] in Mexican neonates. At the genus level, the infant gut microbiota was dominated by *Escherichia-Shigella* spp. and *Bifidobacterium* spp., which aligns with observations by Barker-Tejeda et al.[45] in Spanish infants. However, Chu et al. [46] and Williams et al.[28] reported *Bacteroides* spp. and *Escherichia-Shigella* spp. as the two most dominant genera. The abundance of *Bifidobacterium* spp. in our population was lower than that reported by Ma et al. [47] and Butts et al. [31]. Nonetheless, given that the average age of the infants in our study was approximately 42 days, these findings may reflect an early stage of colonization, characterized by the predominance of Enterobacterales. At this stage, certain facultative anaerobes contribute to reducing oxygen levels in the infant gut, thereby promoting the establishment of strict anaerobes.This biological succession may facilitate the growth of beneficial bacteria such as *Bifidobacterium* spp. [48].

Notably, *Bifidobacterium* spp. and *Escherichia-Shigella* spp. were significantly different between milk and infant samples, but not between milk and the maternal gut microbiome, suggesting an initial transmission route from the maternal gut to milk, which is then partially transferred to the infant. This microbial transmission, along with other milk components such as human milk oligosaccharides (HMOs), may contribute to shaping the infant gut microbiome. *Bifidobacterium* spp. and *Escherichia-Shigella* showed an interesting distribution pattern, with higher abundance in the infant gut compared to milk and the maternal gut. Despite these compositional differences, previous studies have demonstrated vertical transmission species and from the maternal gut to the infant gut microbiome, supporting the existence of an entero-mammary pathway facilitating this microbial transfer [6,7]. Both genera are highly representative in the early-life gut microbiota, the abundance across the mother-infant-milk triad could serve as a microbiome biomarker, guiding interventions ranging from dietary modifications to the administration of bioproducts such as probiotics, prebiotics, and symbiotic during lactation*.*

Other observations, which merit further investigation in future studies, involve bacterial taxa of relevance to current health research. For instance, *Faecalibacterium* spp. was found to be abundant in maternal gut samples, less prevalent in human milk, and absent in the infant gut. This genus is considered a biomarker of intestinal health in adults and is a major butyrate producer [36]. Given that endogenous short-chain fatty acids (SCFAs) production is limited during early life, its presence in the maternal gut may benefit maternal health and contribute to SCFA production. It has been hypothesized that SCFAs can be transferred into breast milk [49], potentially serving as an indirect source of SCFAs for the infant during the first six months of life when gut microbial diversity is still low. As complementary foods are introduced, the infant microbiota becomes more diverse, allowing colonization by butyrate-producing bacteria such as *Faecalibacterium* spp.[50,51].

Our study has limitations. First, its cross-sectional design prevents tracking changes over time or understanding how microbes transfer between maternal, milk, and infant ecosystems. Second, the taxonomic resolution used did not allow for identification at the species level, which might miss specific taxa that are biologically important. Third, not assessing key metabolites like short-chain fatty acids limits our ability to gain a more complete understanding of the potential functional interactions between microbial communities and the host. Finally, psychological and detailed dietary variables were not evaluated, which should be considered in future studies.

The lack of information on the maternal-infant microbiome in countries such as Colombia is an issue that urgently requires attention. Understanding the microbial ecology specific to this population is essential to elucidate its impact on health and nutrition. Moreover, it opens up new perspectives for comprehensive healthcare approaches and food innovation aimed at promoting a healthy microbiome during the earliest stages of life.

In summary, this is the only study conducted in a specific Colombian population to date that has simultaneously analysed the microbiota of mothers, human milk, and exclusively breastfed infants during the first three months postpartum. We observe significant microbial differences between groups in terms of bacterial phyla and genera, richness, and diversity. In this context, the infant microbiome is less diverse and mainly composed of Actinobacteria and Proteobacteria, while human milk and maternal microbiota show greater richness and are dominated by Firmicutes. However, despite these differences, shared taxa were identified, supporting the hypothesis of the entero-mammary pathway and emphasizing the importance of the maternal gut microbiota in breastfeeding women. These findings should be considered in clinical and nutritional care strategies for lactating women, as they may provide an opportunity to promote health in the mother-infant dyad.

## Supporting information

S1 Table Initial and post-filter sequencing reads for each sample. (DOCX)

S1 Fig Non-metric multidimensional scaling (NMDS) graph by delivery type. (TIF)

S2 Fig Differentially abundant phylum across sample types. Statistical significance was assessed using ANCOM-BC. (p < 0.05).
(TIF)

S2 Table Data file. (DOCX)
