# Supplementary material for: Microbial profile along the maternal-infant axis: Early characterization and relationships in breastfeeding women and their newborns in a Colombian population
Source: PLoS One. 2026 Jan 8;21(1):e0340091. doi: 10.1371/journal.pone.0340091 (PMC12782371; doi:10.1371/journal.pone.0340091)
Supplement: S1 Table — (DOCX) [file pone.0340091.s001.docx]

**Table S1.** Initial and post-filter sequencing reads for each sample.

| **Sample-id** | **Input** | **Filtered** | **Percentage of input passed filter** | **Denoised** | **Merged** | **Percentage of input merged** | **Non-chimeric** | **Percentage of input non-chimeric** |
| --- | --- | --- | --- | --- | --- | --- | --- | --- |
| 1-B | 100160 | 76046 | 75.92 | 75391 | 73270 | 73.15 | 8652 | 8.64 |
| 1-M | 111535 | 83988 | 75.3 | 78972 | 62955 | 56.44 | 10860 | 9.74 |
| 2-B | 85697 | 63696 | 74.33 | 63134 | 61199 | 71.41 | 5713 | 6.67 |
| 2-M | 139504 | 112512 | 80.65 | 105677 | 79879 | 57.26 | 10338 | 7.41 |
| 3-B | 109142 | 87625 | 80.29 | 86800 | 84372 | 77.3 | 13351 | 12.23 |
| 3-M | 83028 | 62417 | 75.18 | 59503 | 49397 | 59.49 | 9781 | 11.78 |
| 4-B | 114818 | 88207 | 76.82 | 87094 | 82208 | 71.6 | 6906 | 6.01 |
| 4-M | 100926 | 83930 | 83.16 | 79781 | 67601 | 66.98 | 10645 | 10.55 |
| 5-B | 115325 | 86418 | 74.93 | 85756 | 83353 | 72.28 | 10889 | 9.44 |
| 5-M | 117500 | 94114 | 80.1 | 90228 | 75350 | 64.13 | 11026 | 9.38 |
| 6-B | 70025 | 47886 | 68.38 | 47238 | 43484 | 62.1 | 4848 | 6.92 |
| 6-M | 95262 | 74198 | 77.89 | 70737 | 58797 | 61.72 | 8614 | 9.04 |
| 7-B | 94433 | 64182 | 67.97 | 63422 | 60293 | 63.85 | 9367 | 9.92 |
| 7-M | 95156 | 73724 | 77.48 | 68749 | 51132 | 53.73 | 8183 | 8.6 |
| 8-B | 106943 | 79124 | 73.99 | 78580 | 75880 | 70.95 | 15576 | 14.56 |
| 8-M | 120309 | 102322 | 85.05 | 97873 | 84325 | 70.09 | 16193 | 13.46 |
| 9-B | 100042 | 77947 | 77.91 | 76952 | 74208 | 74.18 | 9959 | 9.95 |
| 9-M | 119185 | 87439 | 73.36 | 85927 | 80343 | 67.41 | 7943 | 6.66 |
| 10-B | 64547 | 48712 | 75.47 | 48328 | 45882 | 71.08 | 3969 | 6.15 |
| 10-M | 115655 | 91767 | 79.35 | 86163 | 68875 | 59.55 | 10621 | 9.18 |
| 11-B | 100217 | 75115 | 74.95 | 74247 | 69266 | 69.12 | 6583 | 6.57 |
| 11-M | 109447 | 88495 | 80.86 | 83076 | 63784 | 58.28 | 9107 | 8.32 |
| 12-B | 106489 | 84274 | 79.14 | 83676 | 82091 | 77.09 | 4737 | 4.45 |
| 12-M | 118392 | 102138 | 86.27 | 98879 | 86476 | 73.04 | 15250 | 12.88 |
| 13-B | 92734 | 68279 | 73.63 | 67088 | 63067 | 68.01 | 7700 | 8.3 |
| 13-M | 90231 | 74608 | 82.69 | 70883 | 61466 | 68.12 | 11518 | 12.77 |
| 14-B | 116866 | 92382 | 79.05 | 91359 | 84461 | 72.27 | 7790 | 6.67 |
| 14-M | 108461 | 81556 | 75.19 | 77995 | 63643 | 58.68 | 10089 | 9.3 |
| 15-B | 96374 | 75790 | 78.64 | 75021 | 73043 | 75.79 | 5848 | 6.07 |
| 15-M | 104781 | 88821 | 84.77 | 85411 | 73576 | 70.22 | 12166 | 11.61 |
| 16-B | 108133 | 82542 | 76.33 | 81777 | 77351 | 71.53 | 9046 | 8.37 |
| 16-M | 113418 | 96561 | 85.14 | 92417 | 79503 | 70.1 | 13402 | 11.82 |
| B-17 | 114906 | 90176 | 78.48 | 89739 | 85760 | 74.63 | 15628 | 13.6 |
| M-17 | 106709 | 79233 | 74.25 | 76241 | 67325 | 63.09 | 8861 | 8.3 |
| B-18 | 94163 | 78341 | 83.2 | 77850 | 76544 | 81.29 | 7482 | 7.95 |
| M-18 | 127580 | 100900 | 79.09 | 99415 | 95465 | 74.83 | 10679 | 8.37 |
| B-19 | 110184 | 86599 | 78.59 | 85835 | 82739 | 75.09 | 7672 | 6.96 |
| M-19 | 111739 | 94447 | 84.52 | 90896 | 80607 | 72.14 | 11631 | 10.41 |
| B-20 | 95296 | 80098 | 84.05 | 79615 | 78309 | 82.17 | 10959 | 11.5 |
| M-20 | 114328 | 88788 | 77.66 | 86941 | 81359 | 71.16 | 10964 | 9.59 |
| B-21 | 119497 | 91840 | 76.86 | 91221 | 86934 | 72.75 | 7816 | 6.54 |
| M-21 | 120791 | 94109 | 77.91 | 89644 | 76562 | 63.38 | 14542 | 12.04 |
| B-22 | 67046 | 49756 | 74.21 | 49141 | 46487 | 69.34 | 5112 | 7.62 |
| M-22 | 90290 | 74373 | 82.37 | 70947 | 60541 | 67.05 | 12048 | 13.34 |
| B-23 | 103198 | 81107 | 78.59 | 80308 | 76427 | 74.06 | 15624 | 15.14 |
| M-23 | 86522 | 71692 | 82.86 | 69923 | 65321 | 75.5 | 17850 | 20.63 |
| B-24 | 104997 | 82370 | 78.45 | 81485 | 79374 | 75.6 | 8703 | 8.29 |
| M-24 | 104076 | 85046 | 81.72 | 83116 | 77683 | 74.64 | 10291 | 9.89 |
| B-25 | 118300 | 85357 | 72.15 | 84909 | 81431 | 68.83 | 6700 | 5.66 |
| M-25 | 90654 | 77224 | 85.19 | 74575 | 64616 | 71.28 | 13932 | 15.37 |
| B-26 | 91570 | 68855 | 75.19 | 68234 | 65677 | 71.72 | 10610 | 11.59 |
| M-26 | 91728 | 77494 | 84.48 | 74517 | 64575 | 70.4 | 12117 | 13.21 |
| B-27 | 111095 | 83680 | 75.32 | 82841 | 80484 | 72.45 | 13760 | 12.39 |
| M-27 | 92800 | 73460 | 79.16 | 68954 | 53772 | 57.94 | 7740 | 8.34 |
| B-28 | 95565 | 74019 | 77.45 | 72988 | 68837 | 72.03 | 5235 | 5.48 |
| M-28 | 102159 | 83031 | 81.28 | 79879 | 71031 | 69.53 | 13142 | 12.86 |
| B-29 | 121703 | 94470 | 77.62 | 93308 | 85223 | 70.03 | 8049 | 6.61 |
| M-29 | 86446 | 67160 | 77.69 | 64613 | 55331 | 64.01 | 8704 | 10.07 |
| B-30 | 113844 | 90092 | 79.14 | 89052 | 84883 | 74.56 | 9474 | 8.32 |
| M-30 | 91122 | 66886 | 73.4 | 63236 | 53337 | 58.53 | 8994 | 9.87 |
| 10L | 88478 | 75737 | 85.6 | 74404 | 71093 | 80.35 | 14329 | 16.19 |
| 11L | 96772 | 84978 | 87.81 | 84248 | 83158 | 85.93 | 21013 | 21.71 |
| 12L | 90956 | 78281 | 86.06 | 77558 | 75722 | 83.25 | 21593 | 23.74 |
| 13L | 95231 | 82338 | 86.46 | 81767 | 80795 | 84.84 | 22257 | 23.37 |
| 14L | 93092 | 80014 | 85.95 | 78958 | 76985 | 82.7 | 21990 | 23.62 |
| 15L | 95981 | 79508 | 82.84 | 78707 | 77249 | 80.48 | 16297 | 16.98 |
| 16L | 96570 | 82899 | 85.84 | 81840 | 79768 | 82.6 | 20644 | 21.38 |
| 17L | 79074 | 66893 | 84.6 | 65860 | 62908 | 79.56 | 14037 | 17.75 |
| 18L | 69513 | 58975 | 84.84 | 57937 | 55577 | 79.95 | 12242 | 17.61 |
| 19L | 82519 | 71853 | 87.07 | 71410 | 70415 | 85.33 | 19614 | 23.77 |
| 1L | 132505 | 110736 | 83.57 | 109638 | 106424 | 80.32 | 18785 | 14.18 |
| 20L | 115856 | 98468 | 84.99 | 97159 | 94325 | 81.42 | 17637 | 15.22 |
| 21L | 78828 | 68787 | 87.26 | 67567 | 64086 | 81.3 | 15644 | 19.85 |
| 22L | 77380 | 66720 | 86.22 | 65559 | 63433 | 81.98 | 17714 | 22.89 |
| 23L | 100264 | 85203 | 84.98 | 84052 | 81732 | 81.52 | 15147 | 15.11 |
| 24L | 84883 | 73890 | 87.05 | 72839 | 70136 | 82.63 | 17362 | 20.45 |
| 25L | 100468 | 86447 | 86.04 | 84328 | 77449 | 77.09 | 10638 | 10.59 |
| 26L | 87431 | 75312 | 86.14 | 73305 | 68986 | 78.9 | 18351 | 20.99 |
| 27L | 134239 | 115252 | 85.86 | 113936 | 110421 | 82.26 | 20149 | 15.01 |
| 28L | 80915 | 71466 | 88.32 | 71012 | 70228 | 86.79 | 17058 | 21.08 |
| 29L | 77609 | 67182 | 86.56 | 66613 | 65667 | 84.61 | 17049 | 21.97 |
| 2L | 109188 | 89874 | 82.31 | 88152 | 85842 | 78.62 | 17589 | 16.11 |
| 30L | 103062 | 88233 | 85.61 | 87373 | 85893 | 83.34 | 20146 | 19.55 |
| 3L | 100721 | 82010 | 81.42 | 81650 | 80956 | 80.38 | 14598 | 14.49 |
| 4L | 95504 | 81071 | 84.89 | 80538 | 79487 | 83.23 | 17217 | 18.03 |
| 5L | 111428 | 95568 | 85.77 | 94081 | 91236 | 81.88 | 17523 | 15.73 |
| 6L | 123615 | 105464 | 85.32 | 105015 | 103713 | 83.9 | 19056 | 15.42 |
| 7L | 148449 | 127036 | 85.58 | 126493 | 125080 | 84.26 | 23225 | 15.65 |
| 8L | 110048 | 95995 | 87.23 | 95250 | 93039 | 84.54 | 11401 | 10.36 |
| 9L | 80397 | 67668 | 84.17 | 66928 | 66084 | 82.2 | 11634 | 14.47 |
