# Supplementary material for: Microbial profile along the maternal-infant axis: Early characterization and relationships in breastfeeding women and their newborns in a Colombian population
Source: PLoS One. 2026 Jan 8;21(1):e0340091. doi: 10.1371/journal.pone.0340091 (PMC12782371; doi:10.1371/journal.pone.0340091)
Supplement: S2 Table — (DOCX) [file pone.0340091.s004.docx]

**Table S3.** Data file

| **Sample**  **id** | **Type** | **Age** | **Previous**  **pregnancies** | **Weight** | **Height** | **BMI** | **BMIclass** | **Fe** | **B9** | **Gestational**  **age** | **Delivery** | **Newborn**  **sex** | **Birth**  **weight** | **Birth_**  **length** | **Breastfeeding**  **First** |
| --- | --- | --- | --- | --- | --- | --- | --- | --- | --- | --- | --- | --- | --- | --- | --- |
| 1260-1M | Mother | 20 | 0 | 55.7 | 1.545 | 23.3 | Adequate | Yes | Yes | 40 | Vaginal | Male | 3230 | 50 | Yes |
| 1260-1B_1 | Baby | 20 | 0 | 55.7 | 1.545 | 23.3 | Adequate | Yes | Yes | 40 | Vaginal | Male | 3230 | 50 | Yes |
| 1L | Milk | 20 | 0 | 55.7 | 1.545 | 23.3 | Adequate | Yes | Yes | 40 | Vaginal | Male | 3230 | 50 | Yes |
| 1260-2M | Mother | 25 | 2 | 48.3 | 1.55 | 20.1 | Adequate | Yes | Yes | 39 | Vaginal | Male | 3700 | 51 | Yes |
| 1260-2B | Baby | 25 | 2 | 48.3 | 1.55 | 20.1 | Adequate | Yes | Yes | 39 | Vaginal | Male | 3700 | 51 | Yes |
| 2L | Milk | 25 | 2 | 48.3 | 1.55 | 20.1 | Adequate | Yes | Yes | 39 | Vaginal | Male | 3700 | 51 | Yes |
| 1260-3M | Mother | 32 | 2 | 65.6 | 1.634 | 24.6 | Adequate | Yes | Yes | 40 | Vaginal | Female | 3390 | 50 | Yes |
| 1260-3B | Baby | 32 | 2 | 65.6 | 1.634 | 24.6 | Adequate | Yes | Yes | 40 | Vaginal | Female | 3390 | 50 | Yes |
| 3L | Milk | 32 | 2 | 65.6 | 1.634 | 24.6 | Adequate | Yes | Yes | 40 | Vaginal | Female | 3390 | 50 | Yes |
| 1260-4M | Mother | 18 | 0 | 61.7 | 1.6 | 24.1 | Adequate | Yes | Yes | 38 | Vaginal | Female | 2920 | 47 | Yes |
| 1260-4B | Baby | 18 | 0 | 61.7 | 1.6 | 24.1 | Adequate | Yes | Yes | 38 | Vaginal | Female | 2920 | 47 | Yes |
| 4L | Milk | 18 | 0 | 61.7 | 1.6 | 24.1 | Adequate | Yes | Yes | 38 | Vaginal | Female | 2920 | 47 | Yes |
| 1260-5M | Mother | 21 | 0 | 57.2 | 1.57 | 23.2 | Adequate | Yes | Yes | 39 | Vaginal | Female | 3020 | 50 | Yes |
| 1260.5B | Baby | 21 | 0 | 57.2 | 1.57 | 23.2 | Adequate | Yes | Yes | 39 | Vaginal | Female | 3020 | 50 | Yes |
| 5L | Milk | 21 | 0 | 57.2 | 1.57 | 23.2 | Adequate | Yes | Yes | 39 | Vaginal | Female | 3020 | 50 | Yes |
| 1260-6M | Mother | 28 | 2 | 64 | 1.56 | 26.3 | Overweight | Yes | Yes | 39 | Vaginal | Male | 3390 | 49 | Yes |
| 1260-6B | Baby | 28 | 2 | 64 | 1.56 | 26.3 | Overweight | Yes | Yes | 39 | Vaginal | Male | 3390 | 49 | Yes |
| 6L | Milk | 28 | 2 | 64 | 1.56 | 26.3 | Overweight | Yes | Yes | 39 | Vaginal | Male | 3390 | 49 | Yes |
| 1260-7M | Mother | 21 | 2 | 74 | 1.598 | 29 | Overweight | Yes | Yes | 39 | Vaginal | Male | 3980 | 52 | Yes |
| 1260-7B | Baby | 21 | 2 | 74 | 1.598 | 29 | Overweight | Yes | Yes | 39 | Vaginal | Male | 3980 | 52 | Yes |
| 7L | Milk | 21 | 2 | 74 | 1.598 | 29 | Overweight | Yes | Yes | 39 | Vaginal | Male | 3980 | 52 | Yes |
| 1260-8M | Mother | 24 | 1 | 56.3 | 1.52 | 24.4 | Adequate | Yes | Yes | 39 | Vaginal | Female | 3540 | 50 | Yes |
| 1260-8B | Baby | 24 | 1 | 56.3 | 1.52 | 24.4 | Adequate | Yes | Yes | 39 | Vaginal | Female | 3540 | 50 | Yes |
| 8L | Milk | 24 | 1 | 56.3 | 1.52 | 24.4 | Adequate | Yes | Yes | 39 | Vaginal | Female | 3540 | 50 | Yes |
| 009-M | Mother | 26 | 1 | 55 | 1.55 | 22.9 | Adequate | Yes | Yes | 39 | Vaginal | Female | 3070 | 47 | Yes |
| 1260-9B | Baby | 26 | 1 | 55 | 1.55 | 22.9 | Adequate | Yes | Yes | 39 | Vaginal | Female | 3070 | 47 | Yes |
| 9L | Milk | 26 | 1 | 55 | 1.55 | 22.9 | Adequate | Yes | Yes | 39 | Vaginal | Female | 3070 | 47 | Yes |
| PHN-M | Mother | 31 | 0 | 48.9 | 1.58 | 19.6 | Adequate | Yes | Yes | 39 | Cesarean | Female | 3130 | 47 | Yes |
| 1000-10B | Baby | 31 | 0 | 48.9 | 1.58 | 19.6 | Adequate | Yes | Yes | 39 | Cesarean | Female | 3130 | 47 | Yes |
| 10L | Milk | 31 | 0 | 48.9 | 1.58 | 19.6 | Adequate | Yes | Yes | 39 | Cesarean | Female | 3130 | 47 | Yes |
| 1260-11M | Mother | 23 | 0 | 68 | 1.61 | 26.2 | Overweight | Yes | Yes | 39 | Vaginal | Male | 3420 | 51 | Yes |
| 1260-11B | Baby | 23 | 0 | 68 | 1.61 | 26.2 | Overweight | Yes | Yes | 39 | Vaginal | Male | 3420 | 51 | Yes |
| 11L | Milk | 23 | 0 | 68 | 1.61 | 26.2 | Overweight | Yes | Yes | 39 | Vaginal | Male | 3420 | 51 | Yes |
| MF-12-M | Mother | 36 | 3 | 64 | 1.546 | 26.8 | Overweight | Yes | Yes | 40 | Cesarean | Male | 3445 | 53 | Yes |
| MF-12-B | Baby | 36 | 3 | 64 | 1.546 | 26.8 | Overweight | Yes | Yes | 40 | Cesarean | Male | 3445 | 53 | Yes |
| 12L | Milk | 36 | 3 | 64 | 1.546 | 26.8 | Overweight | Yes | Yes | 40 | Cesarean | Male | 3445 | 53 | Yes |
| MF-13-M | Mother | 21 | 1 | 64 | 1.655 | 23.4 | Adequate | Yes | Yes | 39 | Vaginal | Male | 3480 | 50 | Yes |
| MF-13-B | Baby | 21 | 1 | 64 | 1.655 | 23.4 | Adequate | Yes | Yes | 39 | Vaginal | Male | 3480 | 50 | Yes |
| 13L | Milk | 21 | 1 | 64 | 1.655 | 23.4 | Adequate | Yes | Yes | 39 | Vaginal | Male | 3480 | 50 | Yes |
| MF-14-M | Mother | 19 | 2 | 50 | 1.494 | 22.4 | Adequate | Yes | Yes | 38 | Cesarean | Male | 3190 | 50 | Yes |
| MF-14-B | Baby | 19 | 2 | 50 | 1.494 | 22.4 | Adequate | Yes | Yes | 38 | Cesarean | Male | 3190 | 50 | Yes |
| 14L | Milk | 19 | 2 | 50 | 1.494 | 22.4 | Adequate | Yes | Yes | 38 | Cesarean | Male | 3190 | 50 | Yes |
| MF-15-M | Mother | 38 | 1 | 63 | 1.53 | 26.9 | Overweight | Yes | Yes | 40 | Vaginal | Female | 3265 | 49 | Yes |
| MF-15-B | Baby | 38 | 1 | 63 | 1.53 | 26.9 | Overweight | Yes | Yes | 40 | Vaginal | Female | 3265 | 49 | Yes |
| 15L | Milk | 38 | 1 | 63 | 1.53 | 26.9 | Overweight | Yes | Yes | 40 | Vaginal | Female | 3265 | 49 | Yes |
| MF-16-M | Mother | 36 | 2 | 71.2 | 1.55 | 29.6 | Overweight | Yes | Yes | 38 | Cesarean | Male | 3080 | 50 | Yes |
| MF-16-B | Baby | 36 | 2 | 71.2 | 1.55 | 29.6 | Overweight | Yes | Yes | 38 | Cesarean | Male | 3080 | 50 | Yes |
| 16L | Milk | 36 | 2 | 71.2 | 1.55 | 29.6 | Overweight | Yes | Yes | 38 | Cesarean | Male | 3080 | 50 | Yes |
| M-17 | Mother | 21 | 0 | 54.5 | 1.61 | 21 | Adequate | Yes | Yes | 37 | Vaginal | Male | 3280 | 49 | Yes |
| B-17 | Baby | 21 | 0 | 54.5 | 1.61 | 21 | Adequate | Yes | Yes | 37 | Vaginal | Male | 3280 | 49 | Yes |
| 17L | Milk | 21 | 0 | 54.5 | 1.61 | 21 | Adequate | Yes | Yes | 37 | Vaginal | Male | 3280 | 49 | Yes |
| M-18 | Mother | 24 | 0 | 56.9 | 1.53 | 24.3 | Adequate | Yes | Yes | 40 | Vaginal | Male | 3350 | 53 | No |
| B-18 | Baby | 24 | 0 | 56.9 | 1.53 | 24.3 | Adequate | Yes | Yes | 40 | Vaginal | Male | 3350 | 53 | No |
| 18L | Milk | 24 | 0 | 56.9 | 1.53 | 24.3 | Adequate | Yes | Yes | 40 | Vaginal | Male | 3350 | 53 | No |
| M-19 | Mother | 34 | 1 | 55.7 | 1.5 | 24.8 | Adequate | Yes | Yes | 38 | Vaginal | Female | 3680 | 48 | Yes |
| B-19 | Baby | 34 | 1 | 55.7 | 1.5 | 24.8 | Adequate | Yes | Yes | 38 | Vaginal | Female | 3680 | 48 | Yes |
| 19L | Milk | 34 | 1 | 55.7 | 1.5 | 24.8 | Adequate | Yes | Yes | 38 | Vaginal | Female | 3680 | 48 | Yes |
| M-20 | Mother | 20 | 0 | 52 | 1.5 | 23.1 | Adequate | Yes | No | 40 | Cesarean | Male | 3086 | 49 | No |
| B-20 | Baby | 20 | 0 | 52 | 1.5 | 23.1 | Adequate | Yes | No | 40 | Cesarean | Male | 3086 | 49 | No |
| 20L | Milk | 20 | 0 | 52 | 1.5 | 23.1 | Adequate | Yes | No | 40 | Cesarean | Male | 3086 | 49 | No |
| M-21 | Mother | 25 | 1 | 73 | 1.588 | 28.9 | Overweight | Yes | Yes | 38 | Vaginal | Male | 3160 | 49 | Yes |
| B-21 | Baby | 25 | 1 | 73 | 1.588 | 28.9 | Overweight | Yes | Yes | 38 | Vaginal | Male | 3160 | 49 | Yes |
| 21L | Milk | 25 | 1 | 73 | 1.588 | 28.9 | Overweight | Yes | Yes | 38 | Vaginal | Male | 3160 | 49 | Yes |
| M-22 | Mother | 19 | 0 | 62.1 | 1.62 | 23.7 | Adequate | Yes | Yes | 39 | Vaginal | Female | 3180 | 52 | No |
| B-22 | Baby | 19 | 0 | 62.1 | 1.62 | 23.7 | Adequate | Yes | Yes | 39 | Vaginal | Female | 3180 | 52 | No |
| 22L | Milk | 19 | 0 | 62.1 | 1.62 | 23.7 | Adequate | Yes | Yes | 39 | Vaginal | Female | 3180 | 52 | No |
| M-23 | Mother | 23 | 1 | 65.8 | 1.565 | 26.9 | Overweight | Yes | Yes | 38 | Vaginal | Male | 3515 | 52 | Yes |
| B-23 | Baby | 23 | 1 | 65.8 | 1.565 | 26.9 | Overweight | Yes | Yes | 38 | Vaginal | Male | 3515 | 52 | Yes |
| 23L | Milk | 23 | 1 | 65.8 | 1.565 | 26.9 | Overweight | Yes | Yes | 38 | Vaginal | Male | 3515 | 52 | Yes |
| M-24 | Mother | 23 | 0 | 55.6 | 1.66 | 20.2 | Adequate | Yes | Yes | 40 | Vaginal | Male | 3130 | 49 | Yes |
| B-24 | Baby | 23 | 0 | 55.6 | 1.66 | 20.2 | Adequate | Yes | Yes | 40 | Vaginal | Male | 3130 | 49 | Yes |
| 24L | Milk | 23 | 0 | 55.6 | 1.66 | 20.2 | Adequate | Yes | Yes | 40 | Vaginal | Male | 3130 | 49 | Yes |
| M-25 | Mother | 34 | 1 | 71.7 | 1.645 | 26.5 | Overweight | Yes | Yes | 40 | Vaginal | Male | 3600 | 51 | Yes |
| B-25 | Baby | 34 | 1 | 71.7 | 1.645 | 26.5 | Overweight | Yes | Yes | 40 | Vaginal | Male | 3600 | 51 | Yes |
| 25L | Milk | 34 | 1 | 71.7 | 1.645 | 26.5 | Overweight | Yes | Yes | 40 | Vaginal | Male | 3600 | 51 | Yes |
| M-26 | Mother | 29 | 1 | 59.1 | 1.62 | 22.5 | Adequate | Yes | Yes | 37 | Cesarean | Male | 2895 | 49 | Yes |
| B-26 | Baby | 29 | 1 | 59.1 | 1.62 | 22.5 | Adequate | Yes | Yes | 37 | Cesarean | Male | 2895 | 49 | Yes |
| 26L | Milk | 29 | 1 | 59.1 | 1.62 | 22.5 | Adequate | Yes | Yes | 37 | Cesarean | Male | 2895 | 49 | Yes |
| M-27 | Mother | 26 | 1 | 62.5 | 1.51 | 27.4 | Overweight | Yes | Yes | 40 | Vaginal | Male | 3560 | 51 | Yes |
| B-27 | Baby | 26 | 1 | 62.5 | 1.51 | 27.4 | Overweight | Yes | Yes | 40 | Vaginal | Male | 3560 | 51 | Yes |
| 27L | Milk | 26 | 1 | 62.5 | 1.51 | 27.4 | Overweight | Yes | Yes | 40 | Vaginal | Male | 3560 | 51 | Yes |
| M-28 | Mother | 18 | 0 | 64.4 | 1.54 | 27.2 | Overweight | Yes | Yes | 38 | Cesarean | Female | 2710 | 47 | Yes |
| B-28 | Baby | 18 | 0 | 64.4 | 1.54 | 27.2 | Overweight | Yes | Yes | 38 | Cesarean | Female | 2710 | 47 | Yes |
| 28L | Milk | 18 | 0 | 64.4 | 1.54 | 27.2 | Overweight | Yes | Yes | 38 | Cesarean | Female | 2710 | 47 | Yes |
| M-29 | Mother | 25 | 0 | 76.2 | 1.61 | 29.4 | Overweight | Yes | Yes | 39 | Vaginal | Male | 3500 | 50 | Yes |
| B-29 | Baby | 25 | 0 | 76.2 | 1.61 | 29.4 | Overweight | Yes | Yes | 39 | Vaginal | Male | 3500 | 50 | Yes |
| 29L | Milk | 25 | 0 | 76.2 | 1.61 | 29.4 | Overweight | Yes | Yes | 39 | Vaginal | Male | 3500 | 50 | Yes |
| M-30 | Mother | 18 | 0 | 48 | 1.545 | 20.1 | Adequate | Yes | Yes | 39 | Vaginal | Female | 3080 | 51 | No |
| B-30 | Baby | 18 | 0 | 48 | 1.545 | 20.1 | Adequate | Yes | Yes | 39 | Vaginal | Female | 3080 | 51 | No |
| 30L | Milk | 18 | 0 | 48 | 1.545 | 20.1 | Adequate | Yes | Yes | 39 | Vaginal | Female | 3080 | 51 | No |
